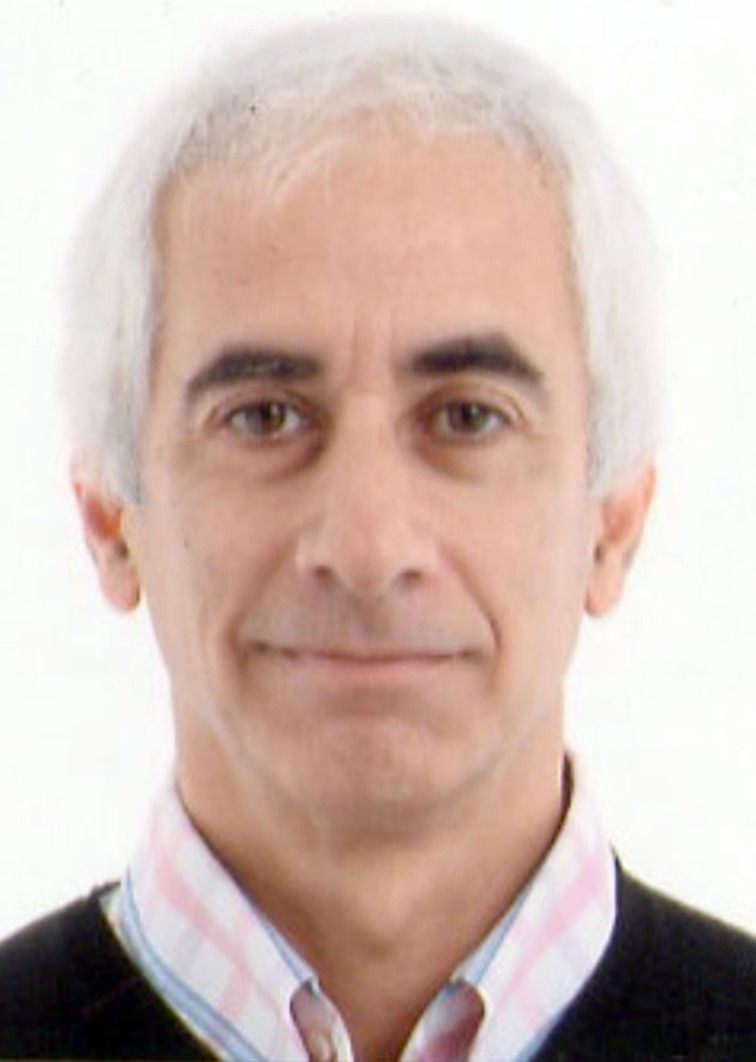# The Coordinate Reaction Model: An Obstacle to Interpreting the Emergence of Chemical Complexity

**DOI:** 10.1002/chem.202101562

**Published:** 2021-08-05

**Authors:** Josep M. Ribó, David Hochberg

**Affiliations:** ^1^ Department of Inorganic and Organic Chemistry Organic Chemistry Section Institute of Cosmos Science (IEEC-UB) University of Barcelona c. Martí i Franquès 1 08028 Barcelona, Catalonia Spain; ^2^ Department of Molecular Evolution Centro de Astrobiología (CSIC-INTA) Ctra. Ajalvir, Km. 4 28850, Torrejón de Ardoz Madrid Spain

**Keywords:** dissipative systems, nonequilibrium processes, reaction mechanisms

## Abstract

The way chemical transformations are described by models based on microscopic reversibility does not take into account the irreversibility of natural processes, and therefore, in complex chemical networks working in open systems, misunderstandings may arise about the origin and causes of the stability of non‐equilibrium stationary states, and general constraints on evolution in systems that are far from equilibrium. In order to be correctly simulated and understood, the chemical behavior of complex systems requires time‐dependent models, otherwise the irreversibility of natural phenomena is overlooked. Micro reversible models based on the reaction‐coordinate model are time invariant and are therefore unable to explain the evolution of open dissipative systems. The important points necessary for improving the modeling and simulations of complex chemical systems are: a) understanding the physical potential related to the entropy production rate, which is in general an inexact differential of a state function, and b) the interpretation and application of the so‐called general evolution criterion (GEC), which is the general thermodynamic constraint for the evolution of dissipative chemical systems.

## Graphical abstract

**Accurate descriptions**: Time‐invariant (T^1^) models are the basis of the molecular description of modern organic chemistry. However, applied to open systems far from equilibrium, they may lead to erroneous interpretations. In such systems, stationary states must be described, in both their stability and evolution (generalized evolution criterion), through irreversibility (time variant, T^−1^) by macroscopic systems methods. Even ordinate, abscissa and curves, in reductionistic graphical descriptions; have different meanings.

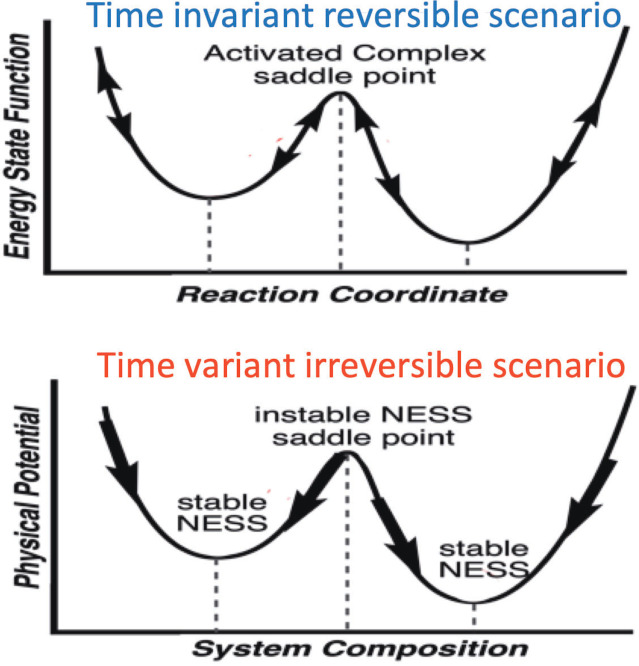

Complex networks of molecules interacting either by noncovalent self‐assembly or through covalent chemical transformations as a whole can lead to emergent properties.[^1^, ^2^, ^3^] A brief look at some examples of systems chemistry reviews^[2]^ shows that their essence, as quoted from Strazewski^[3]^ “is to explore the chemical space of appropriate initial conditions and energy supplies for chemical mixtures to maintain a dynamic state of chemical substances that spontaneously grow in numbers as time goes by.” Implicitly this means that self‐assembly and self‐organization occur in energy dissipative scenarios: systems open to matter and/or to energy exchange, or having inhomogeneous distributions of energy or matter, which belong to the framework of irreversible thermodynamics in the nonlinear regime.

The chemical physics basis of dissipative systems, was established long ago in seminal works, for example, those of Glansdorff, Prigogine, Meixner and Eigen.[^4^, ^5^, ^6^, ^7^] In chemical physics, the topic is of increasing interest and important contributions, with the objective to explain natural chemical systems, are being reported (e. g., ref. [8]). Experimental organic chemists extrapolate the reaction coordinate models of organic chemistry to analyze these scenarios, which belong to irreversible thermodynamics in the nonlinear regime.^[7]^ However, there are a few remarkable exceptions which take into account the role of irreversibility in the reaction dynamics (see, e. g., refs. [10]–[13]). Inadequate descriptions have led to controversies, with the paradigmatic one being the subject of the emergence of spontaneous mirror symmetry breaking (SMSB), that is, obtaining stable biases away from the racemic configuration that are related to the fundamental question of biological homochirality.^[14]^ We discuss here, how a number of such misunderstandings arise from an inappropriate use of organic chemistry chemical models in the irreversibility of dissipative systems.

## Model paradigms in organic chemistry

The foundation of modern chemistry in the 20th century is supported by three pillars:

- The classification in functional groups, whose molecular structures explain physical properties and reactivities, and how the remainder of the molecular backbone may perturb them.^[15]^
- The classification of organic chemical reactions in types according to their mechanisms.[^16^, ^17^] Notice that the mechanisms of organic reactions, nowadays routinely used to discuss actual synthetic results, could have only been achieved using a large arsenal of physical‐chemical techniques and after long experimental work.^[18]^
- The reaction coordinate model (RC) representing an energy state function or relative chemical potentials as functions of the geometrical ordering (distances and angles) between atoms and molecules.^[19]^ It describes the activated complexes at the transition states and reveals the relationship between activation energies (reaction rate constants) and energy state functions (equilibrium constants).[^20^, ^21^] This was proposed by theoretical works,[^22^, ^23^, ^24^] is summarized in the Eyring equation, and was established before the mechanistic models of the former point (b). Furthermore, a paradigm shift followed in the second half of the 20th century when organic chemical versions of the “reaction coordinate”^[19]^ were brought together with the reaction mechanism models, for example, through the Hammond postulate,^[25]^ an organic chemistry concept similar to the older Bell‐Evans‐Polanyi principle.[^26^, ^27^]

The success in the simultaneous use of the three former models converted organic chemistry into a discipline that could be taught and learned easier, and last, but not least, loved easier by chemistry students.^[28]^ One co‐author remembers well, the impact made in 1959 of the surprising and beautiful Cram and Hammond text book,^[29]^ that had broken the tradition^[30]^ to learn organic chemistry through exhaustive memorization of synthetic facts and reaction names.^[31]^ Furthermore, astonishing reports showed that the fate and selectivity of the elementary reactions could be described through the interactions of frontier molecular orbitals,[^30^, ^32^] and that this also explains reactions (pericyclic reactions) which do not follow the Bell‐Evans‐Polany principle.^[33]^ It is difficult to explain today the impact of all this over the past seventy years of the 20th century: each one or two years expert professors should relearn organic chemistry in order to be able to teach it. Finally, a logical consequence of all this, was the use of quantum chemical methods to study reaction paths and the structure of activated complexes.[^34^, ^35^, ^36^, ^37^, ^38^]

## The question of time in chemical models: Reversible and irreversible thermodynamics

Chemical kinetics and RC models, being based on Newtonian mechanics, assume time invariance, that is, time reversal symmetry (microscopic reversibility!). Also, quantum chemistry calculations of reaction paths assume time invariance.^[39]^ This means that the paradigms of organic chemistry do not take into account the origin of time or parity violations nor the cause of the irreversibility of natural phenomena.

Real synthesis involves temperature and mass gradients, that is, an inhomogeneous distribution of matter or energy, and exchange of matter or energy with the surroundings. For example, even a simple flask reflux shows temperature gradients between the heating element and the returning solvent to the solution.^[40]^ Therefore, synthesis mostly belongs to the domain of irreversible thermodynamics, open to matter exchange, or to closed systems which show nonhomogenous energy distributions between the reactant species, as for example in the case of a photochemical reaction. Applied synthesis shows mostly a high variance in yield and selectivity between experiments, because of the lack of control of the interactions with the surroundings. Notice that the principal difference with the time invariant microscopic models, is that applied chemistry deals with macroscopic systems which are under the irreversible nature of time variance (the arrow of time).

**Scheme 1:**
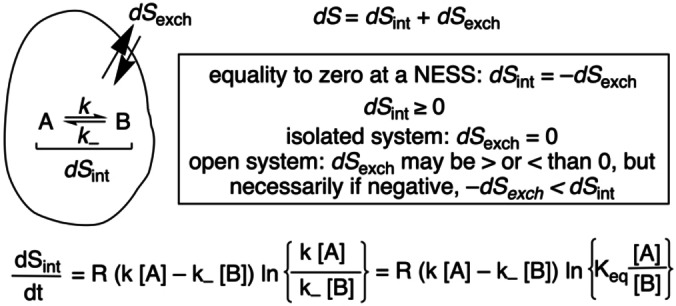
Entropy flows and thermodynamic constraints at any NESS. The internal entropy production for a reaction network is composed of the product of the affinity and the absolute rate in a reversible reaction (see equation at the bottom).^[41]^ Stoichiometric network analysis (SNA)^[42]^ allows one to describe the coupling between reaction network and boundary conditions as a whole, to yield a correct description of the entropy flows of the coupled system (see main text). This discussion is based on ideal systems (activities=1) under isothermal conditions.

The entropy production is always positive for nonequilibrium stationary states, or else is zero at equilibrium. Final stationary states, the so‐called thermodynamically controlled outputs, in an open system, or a closed system exchanging energy with the surroundings, must fulfill the balance between the internal entropy production (d*S*_int_) and the entropy current or exchange (d*S*_exch_) to the surroundings (Scheme 1)

(1) $$P{}_{\mathrm{total}}=P{}_{\mathrm{int}}+P{}_{\mathrm{exch}}=0$$

being d*S/*d*t*=P. Notice, that such nonequilibrium stationary states (NESSs) cannot be described in the framework of reversible thermodynamics, because time irreversibility leads to a permanent and constant rate of entropy production (d*S*_int_ */*d*t*>0) at the final stationary state.

Dissipative systems behavior is determined by the role of the entropy production and entropy exchange. The rate of entropy production is the product of the force by the current, or flow, that it gives rise to. In chemical reactions, the force is the affinity (*Af*) and the current is the absolute reaction rate (Scheme 1):^[41]^ the affinity has, such as expected on physical grounds, a logarithmic relationship with the concentrations, but this is not proportional to the absolute reaction rate. In the *linear* regime of irreversible thermodynamics, *Af* and absolute rates are proportional, and the entropy production at the final stationary state is a minimum, d*P*_int_/d*t*≤0 (theorem of minimum entropy production).[^4^, ^9^] However, despite the historical significance of this, the proportionality between the force and the flux comes only from an expansion of the logarithm near equilibrium,^[9]^ with the requirement that *Af/RT* ≪1, and this means a mathematical approximation that at 300 K, implies *Af*≪2.5 kJ mol^−1^, and so is rarely if ever useful.^[43]^ It is only in this linear range, of limited applied sense, that the theorem of minimum entropy production^[44]^ can be used.

## Evolution of a chemical system when entropy production increases cannot be represented by time invariant models

**Figure 1:**
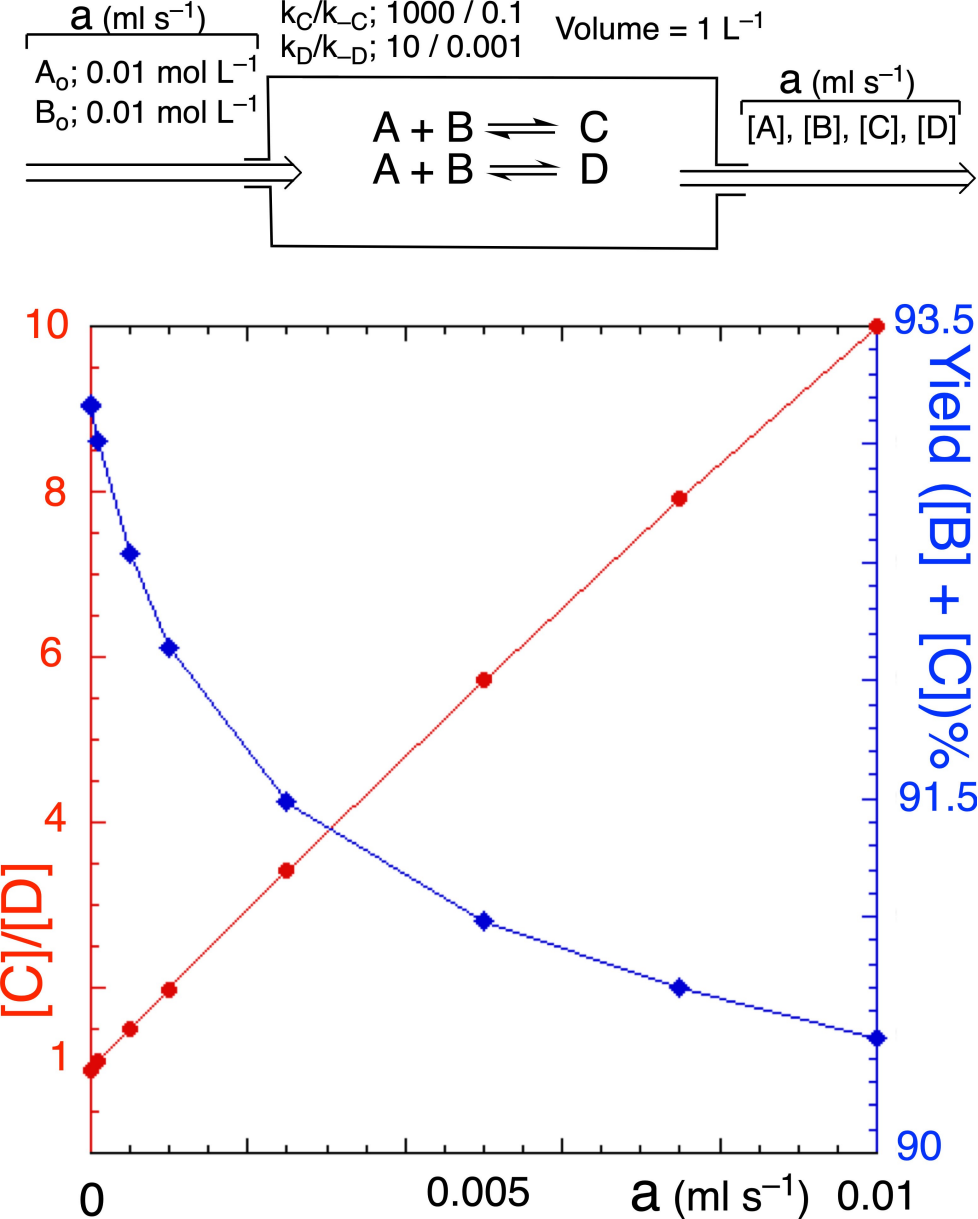
Selectivity ([C]/[D]: red trace) and yields (%; blue trace) for a thermodynamically controlled output (NESS) in an ideal open‐flow reactor as functions of the exchange flow rate (a). As both reactions have the same equilibrium constant, increasing the flow rate increases the selectivity of the reaction with the higher rate constants. However, the effect on the total yield of [C] plus [D] is low. This example points to the advantage of such simple analysis for establishing the system's parameters in the optimization of selectivity (flask reaction synthesis) in open‐flow systems compared to closed ones.

Reaction networks in open systems may be driven, by changing the boundary conditions, toward far from equilibrium states beyond the linear regime. The graphic representations are those of the entropy production with respect to parameters related to the affinity or energy state function (chapter 11, section 11.5. of ref. [4]). When a reaction network is progressively taken away from thermodynamic equilibrium, a final stable NESS is obtained, the so‐called “thermodynamically controlled“ final state. However, in the case of competitive reactions and cycles, the relative composition ratios between species (selectivity) and reaction yields change with the entropy production. In particular, the selectivity might be quite different from that of the thermodynamic equilibrium (see Equation (2) and Figure 1). Notice that the selectivity at the NESS for the simple competitive reaction network in the open system of Figure 1 is given by

(2) $$\frac{\left[C\right]}{\left[D\right]}=\frac{K_{eqC}}{K_{eqD}}exp\left\{\frac{\left({Af}_{D}-{Af}_{C}\right)}{RT}\right\}$$

In reversible thermodynamics, the selectivity is given by the ratio of the equilibrium constants. It is worth noting that when C and D are enantiomers, we not only have equal equilibrium constants but also equal affinities and, therefore, the selectivity ratio must be 1 (racemate) at any final NESS of the thermodynamic branch.

Bistability is an interesting phenomenon appearing in autocatalytic systems showing how kinetic nonlinearities can lead to different NESS's as final states. However, the way to obtain them is path dependent, that is, it depends on the initial conditions of the reaction. The most studied bistable system is the Schlögl model.^[45]^

(3) $$A\overset{\leftarrow }{\to }X\;\;3\;X\overset{\leftarrow }{\to }2\;X+B$$

However, bistability belongs to a system showing hysteresis on the thermodynamic branch and must be considered as a thermodynamically different case from that arising by the destabilization of the thermodynamic branch NESS. Beyond a critical value of the entropy production, the single‐valuedness of the thermodynamic branch, leading to one unique NESS, disappears,^[46]^ and a bifurcation towards different scenarios emerges.

For the thermodynamic branch the slope either remains constant or shows a continuous change. The signature of a bifurcation, this means the instability of the NESS on the thermodynamic branch, is a discontinuity in the slope of the entropy production. Systems of common reactions achieve this critical value of entropy production only at very high concentrations and absolute rates, that leads to the breakdown of the mean field assumption and to diffusion‐controlled reactions. Therefore, the bifurcation in common chemical reactions corresponds to the transit towards inhomogeneous distributions, that may lead to dissipative macroscopic structures. For example, the presence of one diffusion controlled term in the differential rate equations for the Belousov‐Zhabotinsky reaction,^[47]^ leads to an anisotropic matter distribution and to the macroscopic structures characteristic of this reaction.

As the entropy production is the product of the affinity times the absolute rate, the increase of the absolute rate for the same affinity leads to a higher entropy production than that of the uncatalyzed transformation. Furthermore, autocatalysis^[48]^ shows nonlinear kinetic dependences, Therefore, destabilization of the thermodynamic branch and the emergence of new path‐independent NESS's may occur already for ideal solution conditions. Notice, that all reported theoretical networks able to yield multiple NESS's or oscillatory behaviors, that is, to the bifurcation of the thermodynamic branch, belong to autocatalytic systems (see chapter 16 of ref. [4]).

## Potential acting on the evolution of non‐thermodynamic states

One central point in thermodynamics is that concerning the stability of states. The thermodynamic stability criteria (section 18.3. ref. [9]),

(4) $$\frac{d}{dt}\frac{\delta^{2}S}{2}>0$$

(where $\delta^{2}S$ is the second variation of the entropy) applies in reversible and irreversible thermodynamics to the NESS, in both the linear and nonlinear regimes. However, when (4) is negative then the NESS *might be* unstable (p. 410 ref. [9]), but not necessarily. Furthermore, (4) does not distinguish the saddle point condition of the unstable NESS's. In contrast, Jacobian linear stability analysis of the roots of the ordinary kinetic differential equations, and applying the Routh‐Hurwitz criteria, indicate unambiguously the (local) stability or instability of the stationary states.^[9]^

The evolution in a dissipative system is constrained to fulfill the so‐called general evolution criterion (GEC):[^4^, ^5^]

(5) $$d{}_{F}P/dt\leq 0$$

(5) is not an extensive state function, and in general is not an exact differential [*F* stands for the chemical force (affinity)]. This means there is no potential function, whose gradients, with respect to the affinities, yields the indicated temporal derivative of *P*. There is no general way to relate entropy production to affinities, because *P* depends on both affinities (*F*) and the absolute rates (*J*). However, any nonequilibrium state evolves under the constraint (5).

The GEC (5) is the expression of a function related to the rate of dissipated entropy, specific for each system, because it depends on the reactions and system parameters. In the nonlinear regime it may even differ between the different pathways in the compositional hyperspace. Notice that in the linear regime (as well for reversible thermodynamics) because

(6) $$dP/dt=d{}_{F}P/dt+d{}_{J}P/dt$$

and due to the linear relationship between forces and currents,

(7) $$d{}_{F}P/\mathrm{dt}=d{}_{J}P/\mathrm{dt}$$

hence from (5), (6) becomes the total derivative[^4^, ^9^]

(8) dP/dt≤0

In the evaluation, if (8) or (5) can be expressed as exact differentials, that is, if a general potential exists, called the kinetic potential (in order to distinguish it from that of energy state function), it must be considered if the simulation is carried out in the range of local or global potentials (see chapters 10 and 9, respectively, in ref. [4]) as summarized as follows:

- *Local potential*: When we consider small fluctuations around a state, they represent very small affinity differences, therefore, there is linearity between forces and currents (section 16.5. in ref. [9]) and in this short range of fluctuations a local kinetic potential *might* exist.
- *Global potential*: When the composition is significantly different from that of thermodynamic equilibrium, the affinity differences are no longer linear with respect to the absolute rates they give rise to, so that there is generally no single‐valued function able to yield (5) as its exact differential. Notice that this is in agreement with the existence of different pathways under the influence of different potentials depending on the initial conditions of the transformation. Furthermore, the final outcomes might be of different types (e. g., stable and unstable NESS, aperiodic and periodic oscillations, etc.), which may be reached depending on the initial conditions.

To understand the evolution of dissipative systems with respect to the entropy state function and entropy production, is not intuitive. Such as previously quoted, in nonequilibrium thermodynamics these concepts are obscure^[49]^ (sic) and “the question is of course, in the first place, not a mathematical one, but rather conceptual.” In this respect and for chemical reactions, distinctions must be made between the configurational, mixing, rotational and vibrational entropy contributions to the free energy state function, and the entropy dissipated by the process irreversibility.^[50]^ The former terms form part of the set of forces/affinities (free energy) of the reactions, but are not the cause that they give rise to (currents/absolute rates): the dissipated entropy arises from the product of both the forces and the currents. The conceptual difference between both terms, is that one is extensive, but not the other one, and is revealed by the comparison of the units of *S* with those of d*S/*d*t*=*P*, respectively. J K^−1^ mol^−1^ and J K^−1^ s^−1^ (J K^−1^ s^−1^ l^−1^ for the specific entropy production *σ*=*P/V*): S is an energy state function, but *P* is the power of the specific system dissipating entropy.

## Reaction coordinate paths versus evolution plots of dissipative systems

**Figure 2:**
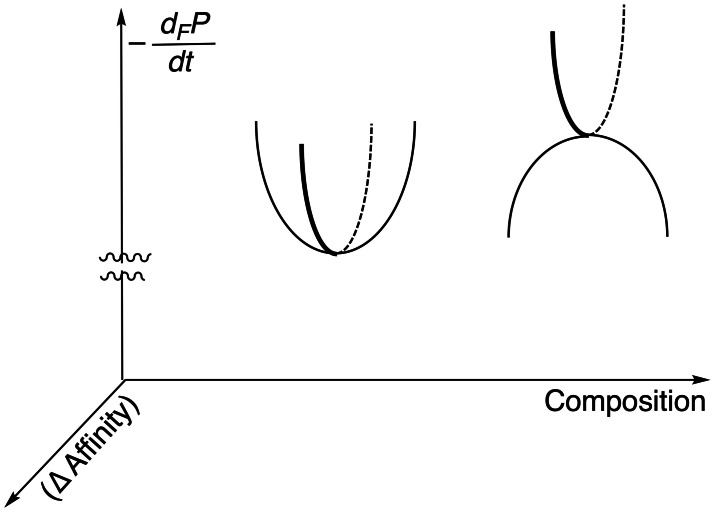
GEC describing, at the local potential scenario, stable and unstable NESSs by simulation of the evolution under small compositional fluctuations. Left: Well potential at a stable NESS. Right: Saddle point potential at an unstable NESS; the normal mode representing the system stability corresponds to compositions retaining the ratio (selectivity) of the reaction products.

When a chemical system has a large number of species, the linear Jacobian stability analysis becomes so involved that it loses practical value. As an alternative, there is the numerical simulation of GEC (5) at a NESS composition by applying very small compositional changes, that is, very small affinity changes (linearity at the local potential range!). This clearly distinguishes between stable NESS's and unstable NESS's:[^42^, ^51^] the stable NESS's are wells of d_*F*_ *P/*d*t* and the unstable NESS are saddle point surfaces (Figure 2). Furthermore, the simulations at the saddle point show that the fluctuations which do not take the system out from the unstable NESS, are those not changing the ratio between the final productive species. For example, in SMSB a fluctuation retaining the racemic composition at the unstable racemic NESS, returns to the unstable racemic NESS:^[50]^ any other type of fluctuation takes the systems towards the stable scalemic NESS (Figure 2, right).

**Figure 3:**
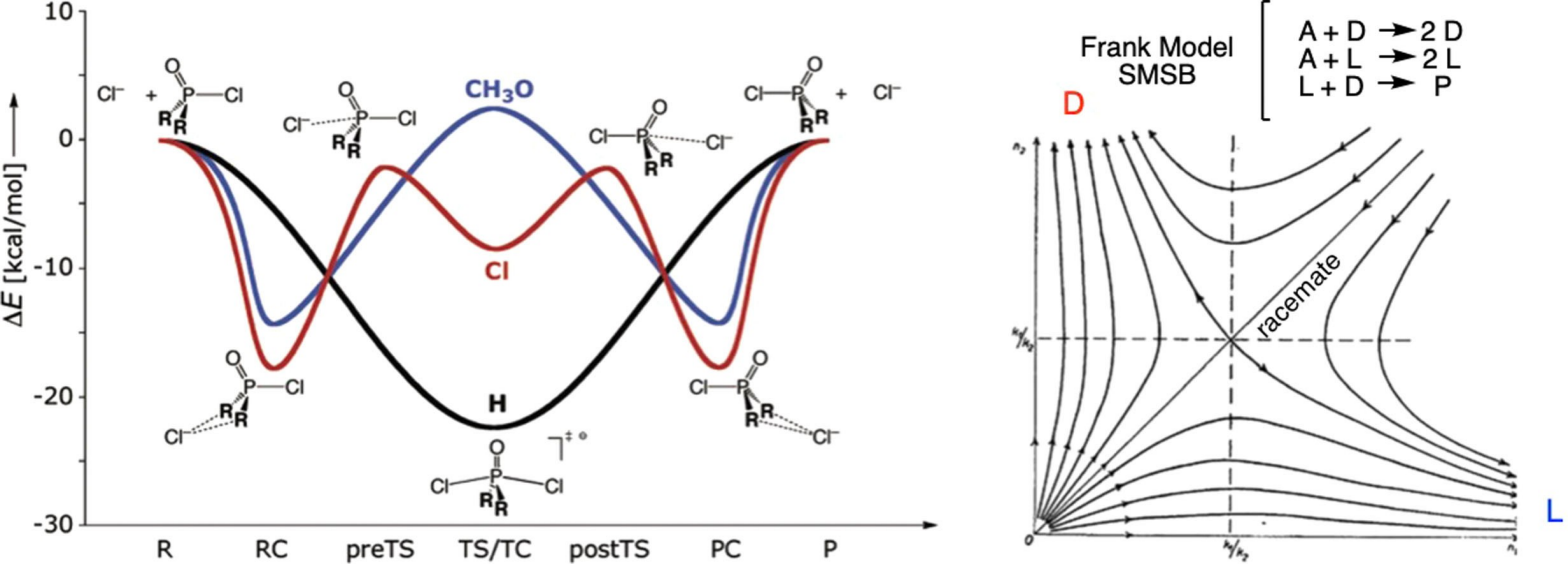
Comparison between a RC plot and one representing irreversible evolution in the compositional space of an energy dissipative system. Left: RC for the S_N_2 substitution at a P=O group from quantum chemical calculations.^[53]^ Right: Attractor representation of SMSB in the Frank model^[54]^ describing the saddle‐point scenario of the racemic instable NESS at the SMSB range. Reproduced with permission from refs. [53] and [54]. Copyright: 2006, American Chemical Society and 1953, Elsevier, respectively.

The above description of potential wells and saddle surfaces is apparently analogous to that of the RC model describing stable compounds and transition states, but such a comparison is misleading. Reaction paths in the RC model are defined by the time reversal invariant microscopic geometrical ordering between atoms in terms of energy state functions (Figure 3 left). However, in dissipative systems representations such as those of Figure 2 describes the energy due to compositional changes of the macroscopic system in respect to a physical potential and the evolution pathways towards the minimum are unidirectional and irreversible. Transformation paths in RC are time reversible (Figure 3, left) and the representation of evolution and stability in macroscopic systems are time variant graphics of attractors (Figure 3, right). Typical graphics of the latter are the Lotka‐Volterra plots in systems biology, which describe the composition of the system in terms of some variable related to *P* or to d_*F*_ *P/*d*t*: there, the potential lines describe NESS's as sources, sinks, saddle points, and the graphic lines represent *unidirectional irreversible* transformations.

All this does not necessarily invalidate RC, which reveals the relationships between reaction rate constants and equilibrium constants, that is, to avoid errors in the consideration of the constraints imposed by the first law of thermodynamics.^[52]^ However the proper description of irreversible evolution in a macroscopic dissipative system should be based on attractor vectorial graphics showing source, sink, and saddle points, such as is done in systems biology.

**Figure 4:**
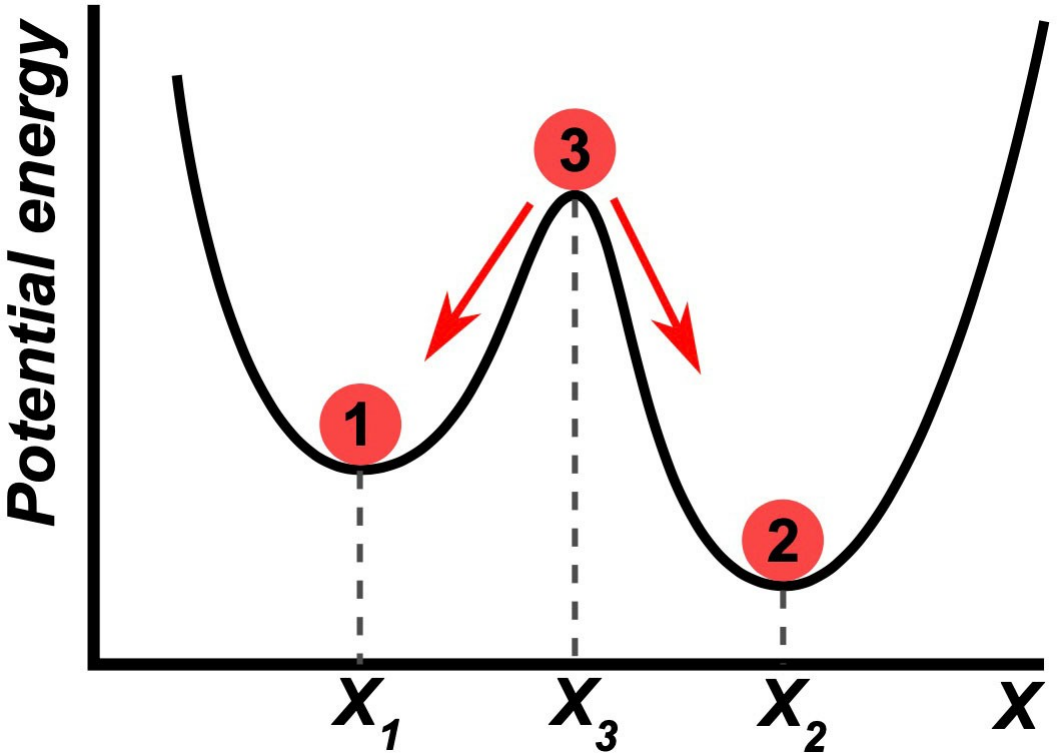
Common qualitative description of the bistability phenomenon. The stable NESSs 1 and 2 are attractors. The system at the unstable NESS 3 and via a compositional fluctuation, depending on the compositional bias, evolves ***irreversibly*** towards one or the other stable NESS. Reproduced with permission from ref. [58]. Copyright: 2020, Wiki Commons.

Attempts to describe graphically the fate of dissipative reaction networks are influenced by the previous extensive use of RC graphical descriptions. In fact, recent reports generally represent dissipative and nondissipative states, in the same figure (i. e., free energy vs. RC graphs), although their authors are well aware of the difference between the dissipative and nondissipative scenarios.[^11^, ^55^] In our opinion, such a representation should be avoided, because in each of the two cases the ordinate and abscissas represent quite different quantities (Figure 4). Specifically, in the time variant irreversible scenario, the ordinate is not related to a thermodynamic energy state function, but to a physical potential, and the abscissa represents changes in composition. By contrast, in the time invariant, thermodynamic reversible scenario, the ordinate is related to the free energy state function and the abscissa to the relative position of atoms along the chemical transformation. Furthermore, the curves representing the transition between states are conceptually different in the two scenarios. In the RC representation, the curves represent the minimum energy path for the achievement of the transformation. In contrast, in the graphical description of a dissipative scenario (Figure 4), the parabolic shaped curves are attractor (irreversible) traces of the transition between states where each point fulfills the constraint of the GEC (5). Figure 4 shows a graphical description of a bistable system:[^56^, ^57^] there are two possible irreversible evolutions (no return path) by a compositional fluctuation from the unstable NESS to either one or the other stable NESS. If the experimental boundary conditions are removed, then the system would evolve according to the reversible thermodynamics scenario, whose representation in RC is different from that occurring in dissipative systems.

*Entropy balance at NESSs*. The internal entropy production and the entropy currents satisfy the entropy balance Equation (1), which must be zero for all NESS where

(9) $$P{}_{\mathrm{total}}=dS/dt;\;P{}_{\mathrm{int}}=dS{}_{\mathrm{int}}/dt;\;P{}_{exch}=dS{}_{\mathrm{exch}}/dt$$

However, (1) is a necessary but not sufficient condition for a NESS. The use of models assuming fixed and constant concentrations of the species exchanged with the surroundings (called clamped scenario) may lead to the erroneous assumption that (1) is a sufficient condition. However, the clamped scenario had its raison d′être in the absence of today's widely available computing facilities, which allow one to find solutions of ordinary differential equation sets and the evolution with time of species concentrations, absolute rates and entropy production currents. Nowadays, there are no valid arguments for using approximations which exclude the matter and energy exchange with the surroundings.[^42^, ^51^, ^59^]

The numerical simulations yield, taking into account the matter flow exchange for the system of Scheme 1, and for a specific flow rate, *P*_int_+*P*_exch_ surfaces, which at the intersection at the zero‐plane surface (*P*=*P*_int_+*P*_exch_=0) yield curves (not singular points!) fulfilling condition (1) (see, e. g., ref. [50]). However, only one or a few singular points of these curves represent an actual NESS: in the case of the system of Scheme 2, only singular points of such a curve, that is, the roots of the differential equation set that fulfill constraint (1). The points must be found either by solving the roots of the ordinary differential equation rates or by the numerical integration of the composition at the final NESS. In these simulations, the results give, with high mathematical precision, the balance of the entropy production with that of exchanging entropy fluxes with the surroundings. In this respect, there are previous reports discussing in more detail the inadequacy of the clamped models.[^42^, ^50^, ^51^, ^57^]

## General evolution criterion as a consequence of time invariance

The GEC (5) has been described as a consequence of the stability criterion.[^4^, ^5^] This, in the sense that the temporal change of the generalized forces proceeds always in a way as to lower the value of the entropy production. We may describe this in a pedagogical argument as follows: the entropy *S* is an increasing function but not continuously increasing: it is the GEC that determines that S tends towards a maximum. It was shown in a recent report,^[60]^ that when the chemical reactions are studied by SNA, the generalization of the GEC for homogeneous well‐mixed systems results as a necessary algebraic condition. Such result arises as a consequence that SNA assumes time variance in the reaction set model, this incorporates irreversibility according to the second principle. In SNA, each individual reaction is represented, also the forward and backward pathways, which are represented as mass action controlled, but irreversible reactions. This corresponds to the real representation of the reaction pathways, that is easily overlooked when the set of reversible reactions is represented by the chemical reaction model that assumes the same reversible pathway (time invariance) for the forward and reverse reactions (micro reversibility). For example, for the “reversible” transformation

(10) $$A\;\overset{\leftarrow }{\to }\;B\;\left(k{}_{1},\;k{}_{m1}\right)$$

the entropy production is given^[41]^ by

(11) $$P{}_{\mathrm{int}}\propto \left(k{}_{1}A-k{}_{m1}B\right)\;\log\left(k{}_{1}A/k{}_{m1}B\right)\geq 0$$

However, when we take into account that forward and backward reactions are different reactions, such as is represented in the SNA stoichiometric matrix,

(12) A→B

(13) B→A

and differ in the probability that they occur, then the force is given by the chemical potentials of the species expressed by the relative chemical potentials,^[42]^ and this leads to

(14) $$P{}_{\mathrm{int}}\;\propto \;(k{}_{1}\left[A\right]\;\log\left\{\left(\left[A\right]/\left[A{}_{\mathrm{eq}}\right]\right)\left(\left[B{}_{\mathrm{eq}}\right]/\left[B\right]\right)\right\}+k{}_{m1}\left[B\right]\;\log\{\left(\left[B\right]/\left[B{}_{\mathrm{eq}}\right]\right)(\left[A{}_{\mathrm{eq}}\right]/\left[A\right])\})\geq 0$$

This is the same expression as in (11), because the equilibrium constant is

(15) $$\left[B{}_{\mathrm{eq}}\right]/\left[A{}_{\mathrm{eq}}\right]=k{}_{1}/k{}_{m1}$$

The use of relative chemical potentials in (14) establishes the relative thermodynamic stability between species, as well the constraint between rate constants and equilibrium constants. However, away from thermodynamic equilibrium the forces in (12) and (13) are never zero and they always create matter flows, which may be missed by looking only at their relative probabilities to determine the values of the equilibrium on expression (11). This is unimportant for a simple reaction as in (10), but in coupled reaction systems the energy partition between different reactions, which are correctly represented by SNA in their elementary currents, determines non‐zero contributions (time invariant currents) to the rate of dissipated entropy. The SNA modelling of (10) decomposing it into (12) and (13) describes the relative sign correlation between force and its effect (matter flow in a reaction), in a similar way that an isotropic temperature gradient determines that heat flows in the sense of higher to lower temperature. Therefore, when we consider the simple reaction network (10) composed by (12) and (13), we must give a relative sign to the forces and currents, but the inner products of force times current, in both individual reactions, will be necessarily positive.^[60]^ Being possible to describe affinities in function of the sum of the products of stoichiometric factors by their flows (see ref. [60]) by SNA, the GEC appears as a necessary algebraic consequence, and arises from the irreversibility of the chemical transformation, this means of time invariance of natural processes. Notice that this concerns not only the nonlinear regime of irreversible thermodynamics, but also NESS in the linear regime and for thermodynamic equilibrium in isolated systems; at the linear regime [Eqs. (7) and (8)] in addition to constraint (1), and in an isolated system without the last constraint, because there is no entropy exchange with the surroundings.

## Concluding Remarks

The behavior of complex chemical networks dissipating energy (e. g., systems open to matter exchange) is under the constraints of the irreversible thermodynamics of the specific macroscopic system. Depending on the boundary conditions, the system dynamics and final states can be quite different for the same reaction mechanisms. Therefore, time‐invariant models such as those commonly used in organic chemistry are insufficient for describing irreversible phenomena.

SNA‐based models and simulations, using working precision above that of machine precision^[61]^ (e. g., the Mathematica package, see, e. g., ref. [62]), are adequate tools for the study of dissipative reaction networks.^[63]^ They can yield not only the evolution with time of the species concentrations, but also that of the entropy currents^[42]^ and of the energetic relationship of the system with the surroundings.^[50]^ Study of the NESSs of autocatalytic reactions in open systems, from an applied point of view, should be based on a comparison of the energy conservation relationships according to the second principle, with yields and selectivity values of the chemical species produced.

## Conflict of interest

The authors declare no conflict of interest.

## Biographical Information

*Josep M. Ribó’s academic career has taken place entirely at the University of Barcelona: he has been a Full Professor of Organic Chemistry since 1989 and emeritus since 2011. In chronological order from 1970, he worked on natural products, oligopyrrole chemistry, polypyrroles as organic conducting polymers, self‐assembly of amphiphilic porphyrins, supramolecular chirality, and spontaneous mirror symmetry breaking in chemical processes*.

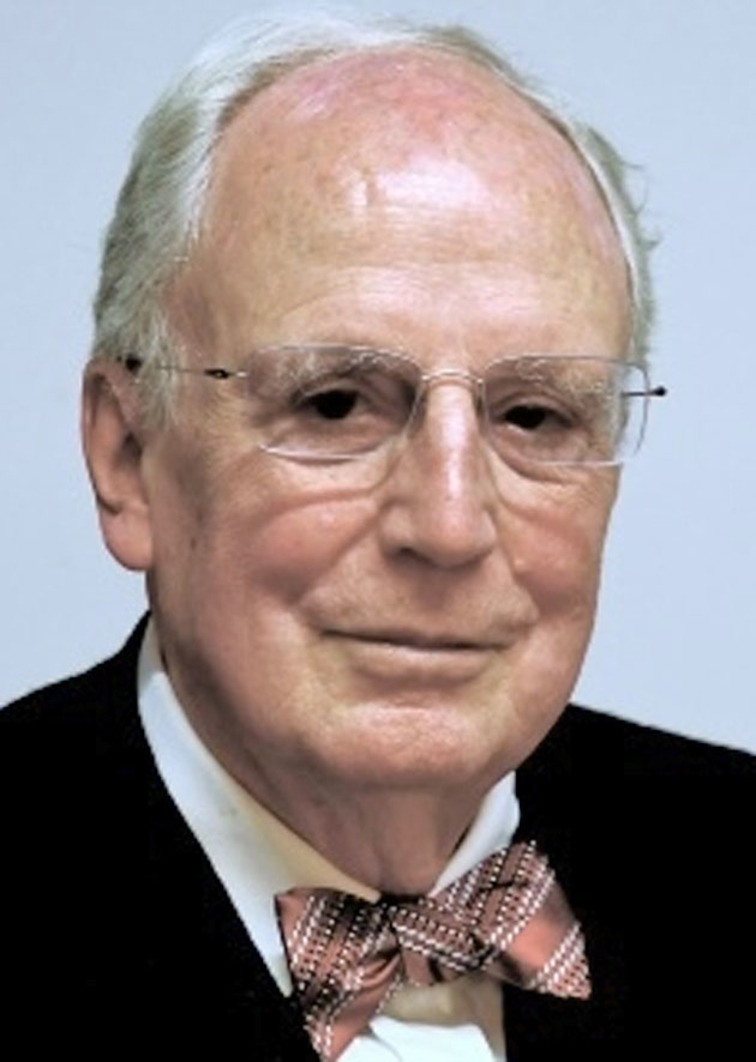

## Biographical Information

*David Hochberg earned a B.A. in physics in 1979 from the University of California, Berkeley, and a Ph.D. in physics from the University of Chicago (1984). After holding postdoctoral positions in physics in the UK, USA, and Spain, he joined the Centro de Astrobiologia (CSIC‐INTA; Madrid) as a founding member, where he is a permanent research scientist. His current research interests include symmetry‐breaking processes in the physics of complex systems and spontaneous mirror symmetry breaking in chemical systems*.